# Electrolyte/Structure-Dependent Cocktail Mediation Enabling High-Rate/Low-Plateau Metal Sulfide Anodes for Sodium Storage

**DOI:** 10.1007/s40820-021-00686-4

**Published:** 2021-08-17

**Authors:** Yongchao Tang, Yue Wei, Anthony F. Hollenkamp, Mustafa Musameh, Aaron Seeber, Tao Jin, Xin Pan, Han Zhang, Yanan Hou, Zongbin Zhao, Xiaojuan Hao, Jieshan Qiu, Chunyi Zhi

**Affiliations:** 1 State Key Lab of Fine Chemicals, Liaoning Key Lab for Energy Materials and Chemical Engineering, School of Chemical Engineering, Dalian University of Technology, Dalian, 116024 P. R. China; 2 Manufacturing, Commonwealth Scientific and Industrial Research Organization (CSIRO), Clayton, VIC 3168 Australia; 3 State Key Laboratory of Fine Chemicals, School of Chemical Engineering, Dalian University of Technology, Dalian, 116024 P. R. China; 4 School of Resources and Environment Engineering, Shandong Agriculture and Engineering University, Jinan, 250100 P. R. China; 5 College of Chemical Engineering, Beijing University of Chemical Technology, Beijing, 100029 China; 6 Department of Materials Science and Engineering, City University of Hong Kong, 83 Tat Chee Avenue, Hong Kong, P. R. China

**Keywords:** Metal sulfide anode, Rate capability, Voltage plateau, Cocktail mediation effect, Sodium-ion batteries

## Abstract

As promising anodes for sodium-ion batteries, metal sulfides ubiquitously suffer from low-rate and high-plateau issues, greatly hindering their application in full-cells. Herein, exemplifying carbon nanotubes (CNTs)-stringed metal sulfides superstructure (CSC) assembled by nano-dispersed SnS_2_ and CoS_2_ phases, cocktail mediation effect similar to that of high-entropy materials is initially studied in ether-based electrolyte to solve the challenges. The high nano-dispersity of metal sulfides in CSC anode underlies the cocktail-like mediation effect, enabling the circumvention of intrinsic drawbacks of different metal sulfides. By utilizing ether-based electrolyte, the reversibility of metal sulfides is greatly improved, sustaining a long-life effectivity of cocktail-like mediation. As such, CSC effectively overcomes low-rate flaw of SnS_2_ and high-plateau demerit of CoS_2_, simultaneously realizes a high rate and a low plateau. In half-cells, CSC delivers an ultrahigh-rate capability of 327.6 mAh g^−1^_anode_ at 20 A g^−1^, far outperforming those of monometallic sulfides (SnS_2_, CoS_2_) and their mixtures. Compared with CoS_2_ phase and SnS_2_/CoS_2_ mixture, CSC shows remarkably lowered average charge voltage up to *ca.* 0.62 V. As-assembled CSC//Na_1.5_VPO_4.8_F_0.7_ full-cell shows a good rate capability (0.05 ~ 1.0 A g^−1^, 120.3 mAh g^−1^_electrode_ at 0.05 A g^−1^) and a high average discharge voltage up to 2.57 V, comparable to full-cells with alloy-type anodes. Kinetics analysis verifies that the cocktail-like mediation effect largely boosts the charge transfer and ionic diffusion in CSC, compared with single phase and mixed phases. Further mechanism study reveals that alternative and complementary electrochemical processes between nano-dispersed SnS_2_ and CoS_2_ phases are responsible for the lowered charge voltage of CSC. This electrolyte/structure-dependent cocktail-like mediation effect effectively enhances the practicability of metal sulfide anodes, which will boost the development of high-rate/-voltage sodium-ion full batteries.

**Supplementary Information:**

The online version contains supplementary material available at 10.1007/s40820-021-00686-4.

## Highlights

- Nano-dispersed SnS_2_ and CoS_2_ phases endow CSC anode with electrolyte/structure-dependent cocktail mediation effect, showing superior rate capability and evidently lowered charge plateau compared with CoS_2_ and SnS_2_/CoS_2_ mixture.
- Alternative electrochemical processes between nano-dispersed different metal sulfides and Na^+^ carriers effectively overcome intrinsic flaws of monometallic sulfide, responsible for the lowered charge plateau of CSC.
- As-assembled CSC//Na_1.5_VPO_4.8_F_0.7_ full-cell shows high-rate capability, and high discharge plateau up to 2.57 V, which is comparable to that with alloy-type anodes.

**Supplementary Information:**

The online version contains supplementary material available at 10.1007/s40820-021-00686-4.

## Graphical abstract

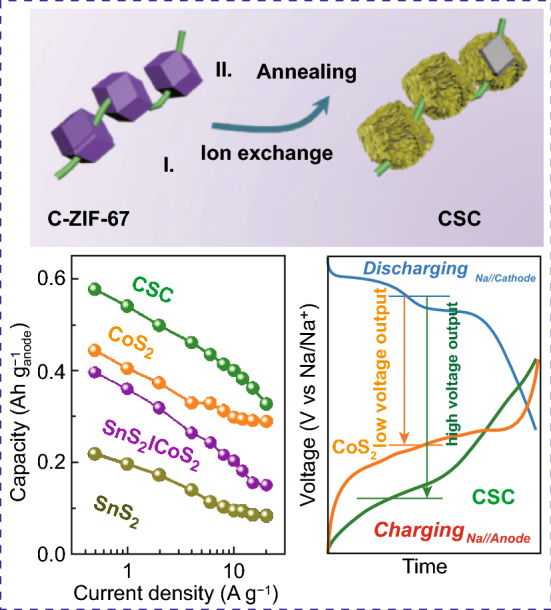

## Introduction

**Scheme 1:**
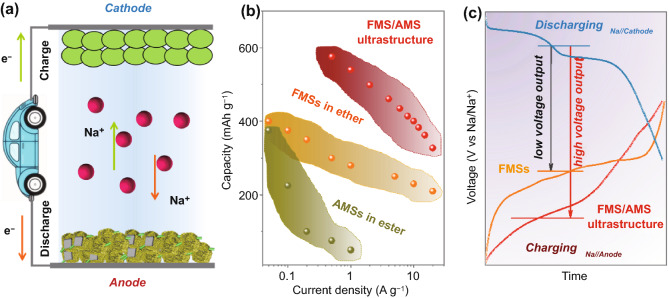
**a** Prototype of full-cells. **b** Rate capability comparison of typical metal sulfide anodes in half-cells. **c** Discharge plateau comparison in full-cells with different metal sulfide anodes showing the merits of FMS/AMS ultrastructure

With the merits of high capacity and low cost, metal sulfides have been recognized as promising anode materials for sodium-ion batteries (SIBs) [1, 2]. However, most metal sulfide anodes examined to date exhibit poor high-rate performance and/or voltage behavior that trends rapidly to relatively high values. The result is full-cells that only operate well at a low rate (≤0.5 A g^−1^_electrode_) and maintain average output voltages typically ≤2 V _ENREF_6 [3–8]. At this level of performance, such cells are only slightly better than a number of advantages in energy density over aqueous batteries (e.g., zinc batteries) but noncomparable safety to the latter [9–12]. Thus far, many studies on metal sulfide anodes still focus on the enhancement in reversible capacity, rate capability, and cyclability in half-cells. Even few studies concerning the properties of metal sulfide anodes in full-cells, most of them only roughly evaluate the performance of full-cells based on anodes instead of total electrodes. This could result in certain intrinsic flaws of metal sulfide anodes underrated [10]. Therefore, from the perspective of full-cell, to solve the low-rate and high-plateau issues of metal sulfide anodes is crucial for the development of high-performance full-cells (Scheme 1a).

Different metal sulfides usually show electrolyte/structure-dependent electrochemical properties, offering valuable inspiration to rationally design new architectures and investigate their properties in proper electrolyte [3, 13, 14]. Compared with ester-based electrolytes, ether-based electrolytes can effectively inhibit3 the shuttle effect of polysulfides in situ formed during discharge/charge processes, thus more beneficial to obtain reversible properties of metal sulfides [1, 13, 15]. Ferromagnetic metal (Fe, Co, Ni, etc.) sulfides (FMSs) are very promising conversion-reaction anode materials widely studied for SIBs [16–19]. Compared with conventional hard carbon or red phosphorous anodes, FMS anodes can display ultrahigh-rate capability (≥20 A g^−1^) in ether-based electrolyte, holding a great promise in SIBs (Scheme 1b) [20–22]. However, FMS anode usually suffers from severe voltage hysteresis and high plateau (~1.9 V vs Na/Na^+^), largely lowering the discharge plateau of full-cells (Scheme 1c). From this point, mono-component FMSs seem to be difficult to meet the requirements for high-performance full-cells. So far, despite many relevant studies, most of them are inclined to ignoring the severe intrinsic flaws of FMSs, emphasizing to enhance capacity and cyclability. By contrast, another series of metal (Sn, Sb, Bi, etc.) sulfides (AMSs) with conversion/alloying-reaction mechanisms can show acceptable voltage hysteresis and relatively lower voltage plateau [23–30]. However, these AMSs always suffer from severe volume change during discharge/charge processes, resulting in poor rate capability and cyclability in ester-based electrolytes (Scheme 1b). Owing to latent catalysis over the decomposition of certain ether, such AMSs remain scarcely investigated in ether-based electrolyte [31, 32]. Encouragingly, by utilizing fluorine-containing sodium salt in ether solvents as electrolyte, the undesirable catalysis of AMSs can be effectively suppressed to allow a stable battery operation [33, 34]. The good compatibility enables the investigation of electrochemical properties of FMS/AMS composites in ether-based electrolytes. In the multi-component metal sulfide anodes, each component functions as active material and mutually compete. Thus, the electrochemical behaviors of multi-component metal sulfides are comprehensive results from individual component. Given that exotic properties beyond rule-of-mixtures (cocktail-like mediation effect) in multi-component high-entropy nano-systems [35, 36], to construct new superstructures assembled by nano-dispersed FMSs and AMSs and to study their properties in ether-based elctrolytes, could be an effective strategy toward high-performance full-cells. Additionally, the poor conductivity of most metal sulfides makes them essential to further combine with highly conductive carbon materials. Such combination can endow rational architectures with fast ion/electron transfer, which is conducive to obtaining satisfactory electrochemical properties [13, 37–39]. So far, despite some studies pertaining to FMS/AMS composites, the certain agglomeration or phase separation between FMS and AMS remains unsatisfactory to investigate their comprehensive impact. Additionally, such studies mostly involved the electrochemical properties of FMS/AMS composites in carbonate-based electrolytes [40–42]. Thus, to study the voltage behavior of metal sulfide composites in ether-based electrolytes will provide a new perspective to pursue desired sodium storage properties.

Herein, CNTs-stringed metal sulfides superstructure anode assembled by nano-dispersed SnS_2_ and CoS_2_ phases (CSC, C: CNT; S: SnS_2_; C: CoS_2_) is engineered to combine the merits of FMS- and AMS-type anode materials, aiming at simultaneously solving the dual-problems of poor rate capability/output-voltage characteristics (Scheme 1b-c). The highly nano-dispersed metal sulfides in CSC show remarkable cocktail-like mediation effect, effectively circumventing intrinsic drawbacks of different metal sulfides. The ether-based electrolyte greatly enhances the reversibility of metal sulfides, which can inhibit the aggregation of homogenous metal sulfides, enabling a long-life effectivity of cocktail-like mediation. In half-cells, CSC delivers an ultrahigh-rate capability of 327.6 mAh g^−1^ at 20 A g^−1^, showing remarkably lowered average charge plateau up to 0.62 V vs Na/Na^+^, compared with CoS_2_ phase and SnS_2_/CoS_2_ mixture. The as-assembled CSC//Na_1.5_VPO_4.8_F_0.7_ full-cell shows a good rate capability (0.05 ~ 1.0 A g^−1^, 120.3 mAh g^−1^_electrode_ at 0.05 A g^−1^) and a high average discharge voltage up to 2.57 V, comparable to full-cells with alloy-type anodes. Kinetics and mechanism studies reveal that the cocktail mediation effect largely boosts the charge transfer and ionic diffusion in CSC; along the diffusion direction of Na^+^ carriers, alternative and complementary electrochemical processes between different nano-dispersed metal sulfides (SnS_2_, CoS_2_) and Na^+^ carriers are responsible for the lowered average charge plateau of CSC. This exhibited cocktail-like mediation effect evidently improves the practicability of metal sulfide anodes, which will boost the development of high-rate/-voltage sodium-ion full batteries.

## Results and Discussion

### Materials Preparation and Characterization

**Fig. 1:**
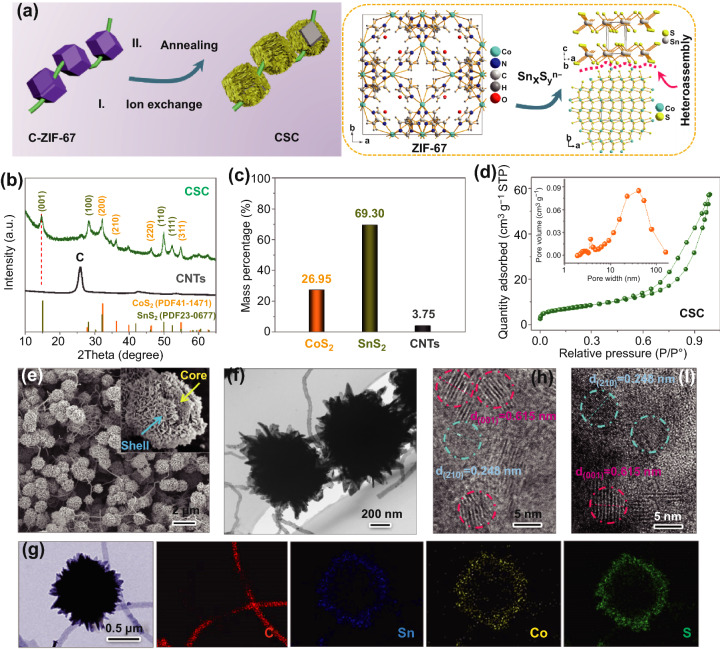
**a** Schematic illustration of fabrication process of CSC, inset (right) showing the reaction between ZIF-67 and Sn_*x*_S_*y*_^n−^. **b** XRD patterns of CNTs and CSC. **c** Mass content of CoS_2_, SnS_2_, and CNTs in the CSC. **d** N_2_ adsorption isotherm of CSC and corresponding pore width distribution. **e** FE-SEM images of CSC (inset displaying the core/shell structure of CSC). **f** TEM image of CSC and **g** TEM-EDS element mapping of CSC including C, Co, Sn, and S. HR-TEM images of **h** shell and **i** core in CSC showing co-assembly of nano-CoS_2_ and -SnS_2_

The CSC was initially obtained by ion-exchange reaction between thiostannate (Sn_x_S_y_^n−^) and cobalt-based zeolitic imidazolate framework (ZIF-67) followed by annealing treatment (Fig. 1a). For an enhanced conductivity of the resulting CSC, the ZIF-67 particles (C-ZIF-67) are connected together (‘stringed’) by a network of CNTs (Fig. S1). Sn^119^ NMR spectroscopy reveals that several tetravalent thiostannate species (SnS_3_^2−^, SnS_4_^4−^, and Sn_2_S_6_^4−^) exist in solution and these are referred to collectively as ‘Sn_x_S_y_^n−^’ (Fig. S2) [43]. Within the ion-exchange process, Co^2+^ in ZIF-67 reacts rapidly with Sn_x_S_y_^n−^ species, forming a unique superstructure comprised of nano-dispersed CoS_2_ and SnS_2_ phases. The overall reaction follows Eq. (1):

1 $$ {\text{Sn}}_{x} {\text{S}}_{y}^{n - } + {\text{Co}}^{2 + } \to {\text{SnS}}_{2} + {\text{CoS}}_{2} \;\;\left( {1 \le x \le 2,\;\;3 \le y \le 6,\;\;2 \le n \le 4} \right) $$

As shown in Fig. 1b, X-ray diffraction (XRD) patterns exhibit the diffraction peaks of CoS_2_ (PDF No. 00-41-1471), SnS_2_ (PDF No. 00-23-0677), and carbon, verifying their presence in the CSC. Compared with standard phase, the reflection for the (0 0 1) plane of SnS_2_ registers a slight shift toward lower angles, implying an expanded interlayer spacing [14, 44]. The expanded interlayer spacing could be associated with the use of thiostannate precursor and low-temperature ion-exchange process. The ion-exchange reaction of thiostannate with ZIF-67 typically occurs at −5 °C in 1 h, where fast reassembly of SnS_2_ results in the expanded interlayer spacing. Also, the relatively low annealing temperature (450 °C) is beneficial to retain the expanded interlayer spacing of SnS_2_. The content of carbon nanotubes in CSC is obtained by thermogravimetric analysis (TGA), which is ca. 3.75 wt% (Fig. S3). By inductively coupled plasma-mass spectrometry (ICP-MS), the elemental content of CSC is analyzed, revealing that the mole ratio of Co/Sn/S is *ca.* 1.00/1.73/5.46 (Table S1). The corresponding mass content of CoS_2_ and SnS_2_ in the CSC is 26.95 and 69.30wt%, respectively (Fig. 1c). The type-IV N_2_ adsorption isotherms of CSC present an evident hysteresis loop, indicating the presence of mesopores (Fig. 1d). The corresponding pore width (inset) mainly centers in the range of 20–45 nm. The theoretical capacity of CSC anode (C_*T*-CSC_) can be evaluated roughly according to the equation: C_*T*-CSC_ = *x*C_*T*-CoS2_ + *y*C_*T*-SnS2_, where *x* and *y* is the percentage content of CoS_2_ and SnS_2_ in the CSC. The C_*T*-CoS2_ and C_*T*-SnS2_ are the theoretical capacity of CoS_2_ and SnS_2_, which is 872 and 1136 mAh g^−1^, respectively. Thus, C_*T*-CSC_ = 0.2695 × 872 + 0.695 × 1136 = 1024.5 mAh g^−1^.

Figure 1e exhibits field emission scanning electron microscopy (FE-SEM) images of CSC, which consists of carbon nanotubes-stringed core/shell architecture (inset). Such core/shell structures are greatly influenced by precursors, solvents, reaction temperatures, and concentrations (Figs. S4–S6). The content of SnS_2_ in the CSC can be tuned to some extent by varying the concentration of thiostannate solution (Fig. S6). Transmission electron microscope (TEM) image shows the typical radial morphology of the CSC (Fig. 1f). Energy-dispersive spectrometer (EDS) elemental mapping yields a distribution of the elements C, Sn, Co, and S in the CSC, which correspond well with the TEM image (Fig. 1g). The details of shell and core were further characterized by TEM. The shell is actually composed of nanosheets (Fig. S7a). As displayed in Fig. 1h, high-resolution transmission electron microscope (HR-TEM) image clearly exhibits interplanar spacings of 0.248 and 0.615 nm for CoS_2_ (2 1 0) and SnS_2_ (0 0 1) lattice planes, verifying such nanosheets assembled by nano-dispersed SnS_2_ (red) and CoS_2_ (blue-green). The TEM-EDS line-scan profiles show matched peaks with Co, Sn, S elements, further suggesting the superstructure of shell co-assembled by SnS_2_ and CoS_2_ phases (Fig. S7b). Corresponding to SEM image of CSC (inset), the core of CSC shows an abundant microstructure, in which the pore (green) can be observed (Fig. S8a). As shown in Fig. 1i, HR-TEM image of the core also exposes the lattice planes of SnS_2_ (0 0 1) and CoS_2_ (2 1 0), which accord with the corresponding selected area electron diffraction (SAED) pattern (Fig. S8b). Such results verify that the core of CSC is also assembled by nano-dispersed SnS_2_ and CoS_2_ phases. The CSC was further analyzed by X-ray photoelectron spectroscopy (XPS). As shown in Fig. S9, compared with commercial CoS_2_ sample, the high-resolution of XPS of Co 2p of CSC shows a *ca.* 0.45 eV shift toward higher binding energy. Moreover, the high-resolution of XPS of Sn 3d of CSC also appears a 0.61 eV shift toward higher binding energy. Such results imply the presence of chemical effect between CoS_2_ and SnS_2_ in CSC anodes [45, 46].

### Half-Cell Properties

**Fig. 2:**
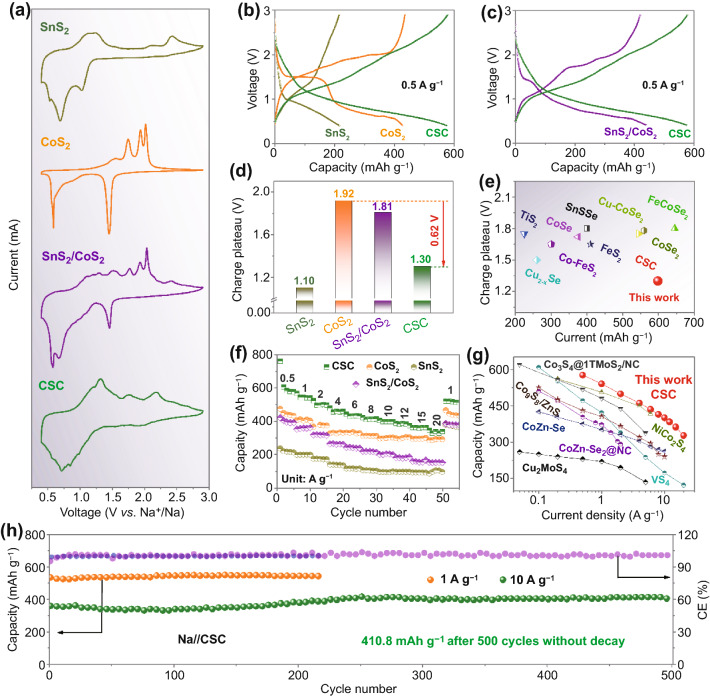
**a** CV curves and **b, c** corresponding discharge–charge curves of CSC, commercial SnS_2_ and CoS_2_, and SnS_2_/CoS_2_ mixture. **d** Histogram showing the average charge plateau voltages of various anodes in half-cells. **e** Capacity/charge plateau comparison of different anodes. **f** Rate capability of CSC, commercial SnS_2_ and CoS_2_ in half-cells. **g** Rate capability comparison of different anodes. **h** Long-life cyclability of CSC anode at 1 and 10 A g^−1^ (*CE* Coulombic efficiency)

The electrochemical properties of anode materials are firstly evaluated by testing half-cells with Na foil as counter electrode and ether-based electrolytes with fluorine-containing sodium salt. For comparison, commercial SnS_2_ and CoS_2_ powders with well-matched XRD patterns to standard phases are also tested (Fig. S10). Compared with the CSC, the N_2_ isotherms of commercial SnS_2_ and CoS_2_ samples typically exhibit no evident hysteresis loop, whereby the corresponding pore diameter distributions display nonporous properties (Fig. S11). After initial three scans at 0.1 mV s^−1^, mono-component metal sulfides (CoS_2_ and SnS_2_) and anodes composed of both compounds show gradually stabilized CV curves (Fig. S12). The initial CV curve of the CSC anode shows three oxidation peaks, which are associated with SnS_2_ phase at 0.70–1.55 V and CoS_2_ phase at 1.70–2.10 V, respectively. The reduction peak at 1.60–1.80 V is correlated with the CoS_2_ phase, while the peaks at 0.50–1.10 V are linked to SnS_2_ and formation of solid electrolyte interphase (Fig. S13a). In subsequent scans, the reduction peak related to CoS_2_ gradually disappears, which could result from electrochemical activation of nano-dispersed SnS_2_ and CoS_2_ phases [16, 22]. As shown in Fig. 2a, the activated CSC delivers a main oxidized peak potential range (0.75–1.65 V), which is close to that of SnS_2_ (0.80–1.45 V) but remarkably lower than that of CoS_2_ (1.30–2.18 V) and SnS_2_/CoS_2_ mixture (1.25–2.15 V). Correspondingly, CSC anode displays an average charge voltage of *ca.* 1.30 V, which is close to that of SnS_2_ but lower than that of CoS_2_ (*ca.* 1.92 V) (Fig. 2b). Compared with commercial SnS_2_/CoS_2_ mixture with average charge voltage of *ca.* 1.81 V, CSC anode also shows evident low-plateau merit (Fig. 2c). This verifies that the construction of a superstructure assembled from nano-dispersed SnS_2_ and CoS_2_ phases is crucial for lowering the intrinsically high plateau of the CoS_2_ phase. Specifically, as shown in Fig. 2d, the introduction of nano-dispersed SnS_2_ phase into CSC effectively lowers the intrinsic average charge voltage of CoS_2_ up to *ca.* 0.62 V. This in turn will translate to a higher plateau voltage for full-cells, thereby improving their energy density.

Compared with other metal chalcogenide anodes, CSC exhibits obvious high-capacity and low-plateau advantages (Fig. 2e). Moreover, compared with commercial SnS_2_ and CoS_2_, and mixtures of the two, CSC shows a remarkably improved rate capability, ranging from 0.5 to 20 A g^−1^ with a high capacity of 327.6 mAh g^−1^_anode_ at 20 A g^−1^ (Fig. 2f). The corresponding discharge/charge curves are exhibited in Fig. S14. When tested with ester-based electrolyte, CSC shows similar CV curves to that in ether-based electrolyte, but the reversible capacity, to the same cutoff voltage, shrinks markedly (Fig. S15). In addition, compared with in ether-based electrolyte, the rate capability of CSC is greatly deteriorated (Fig. S16), along with an increased resistance of charge transfer (Fig. S17). Such phenomena suggest the key role of ether-based electrolyte in stabilizing metal sulfide anodes and realizing fast charge transfer, which could be associated with good compatibility between metal sulfide and ether solvent [1, 15]. Evidently, the CSC anode effectively circumvents the intrinsic high voltage of CoS_2_ and low-rate drawback of SnS_2_ in ether-based electrolyte. Compared with other anode materials in half-cells, CSC also shows a remarkable high-rate capability (Fig. 2g, Table S2)_ENREF_12_ENREF_13_ENREF_14_ENREF_15_ENREF_16_ENREF_17_ENREF_18 [47–54]. The CSC can be cycled at high current densities (1 and 10 A g^−1^) with excellent long-life cyclability, specifically, 410.8 mAh g^−1^_anode_ at 10 A g^−1^ over 500 cycles without decay (Fig. 2h).

### Electrochemical Kinetics

**Fig. 3:**
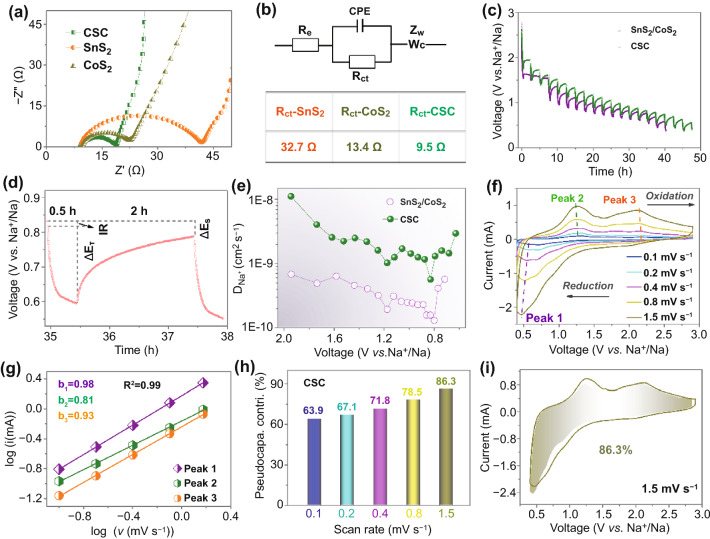
**a** Nyquist plots of different anodes in half-cells and **b** corresponding equivalent circuit and charge transfer resistance (*R*_ct_). **c** GITT profiles of Na//CSC half-cell discharged and **d** typical profile in a single GITT test. **e** Na^+^ diffusion coefficient distribution corresponding to a typical discharge curve of Na//CSC half-cell (inset). **f** CV curves of Na//CSC half-cell at different scan rates. **g** *b*-values obtained by fitting peak current*-*scan rate correlation based on CV curves of Na//CSC half-cell. **h** Pseudocapacitive contribution (pseudocapa. contri.) of Na//CSC at different scan rates. **i** CV profiles of Na//CSC at 1.5 mV s^−1^ and corresponding pseudocapacitive contribution (shaded region)

The electrochemical kinetics of the CSC anode in half-cells is studied in detail by reference to the results of electrochemical impedance spectroscopy (EIS). Compared with electrodes made from commercial samples of SnS_2_ and CoS_2_, the Nyquist curve for a typical CSC anode shows a semi-circle with smaller diameter, implying a faster charge transfer (Fig. 3a). Based on the derived equivalent circuit, the resistances of charge transfer for CSC, commercial SnS_2_ and CoS_2_ anodes are 9.5, 32.7, and 13.4 Ω, respectively (Fig. 3b). To compare Na^+^ diffusion coefficient ($D_{{{\text{Na}}^{ + } }}$) in CSC and SnS_2_/CoS_2_ mixture, galvanostatic intermittent titration technique (GITT) was conducted at 0.05 A g^−1^ for 0.5 h, followed by relaxation for 2 h. The typical GITT discharge profiles of CSC and SnS_2_/CoS_2_ mixture are shown in Fig. 3c. As illustrated in Fig. 3d, $D_{{{\text{Na}}^{ + } }}$ can be calculated following equation $D_{{{\text{Na}}^{ + } }} = \frac{{4L^{2} }}{\pi \tau }\left( {\frac{{\Delta E_{S} }}{{\Delta E_{t} }}} \right)^{2}$, where *L* is Na^+^ diffusion length (cm), *τ* is the current impulse time (s), *t* is relaxation time (s), Δ*E*_*S*_ is steady-state potential change (V), Δ*E*_*t*_ is the instantaneous potential change (V) used to deduce IR drop [55, 56]. Corresponding to the GITT profiles, the calculated average $D_{{{\text{Na}}^{ + } }}$ is *ca.* 0.5 × 10^–9^ cm^2^ s^−1^, which is around twice that in half-cell with SnS_2_/CoS_2_ mixture (Fig. 3e). Evidently, compared with simply mixed SnS_2_/CoS_2_ anode, the CSC assembly of nano-dispersed SnS_2_ and CoS_2_ particles shows remarkable superiority in terms of charge transfer kinetics and ionic diffusion.

Next, the pseudocapacitive contribution to charge storage in the Na//CSC half-cell was evaluated, on the basis that this component gives rise to faster charge transfer kinetics. CV curves at different rates are shown in Fig. 3f, and the correlation of peak currents (*i*) and scan rates (*v*) was assessed against the relationship *i* = *av*^*b*^, where *a* and *b* are adjustable constants [57]. As shown in Fig. 3g, the resultant *b*-values are 0.98, 0.81, and 0.93, respectively, which implies the presence of a substantial pseudocapacitive contribution. The latter can be quantified through the equation *i* = *k*_1_*v* + *k*_2_*v*^1/2^, where *k*_1_*v* and *k*_2_*v*^1/2^ represent pseudocapacitive and ion-diffusion controlled contribution, respectively [57–59]. As shown in Fig. 3h, CSC anodes exhibit dominant pseudocapacitive contributions at scan rates of 0.1, 0.2, 0.4, 0.8, and 1.5 mV s^−1^, specifically, 64.0%, 67.0%, 71.6%, 78.3%, and 86.3%, respectively. Figure 3i displays the CV curves of Na//CSC at 1.5 mV s^−1^, in which the shaded region represents the pseudocapacitive contribution. This, together with the small charge transfer resistance and high $D_{{{\text{Na}}^{ + } }}$, explains the excellent rate capability of the CSC anode.

### Electrochemical Mechanism

**Fig. 4:**
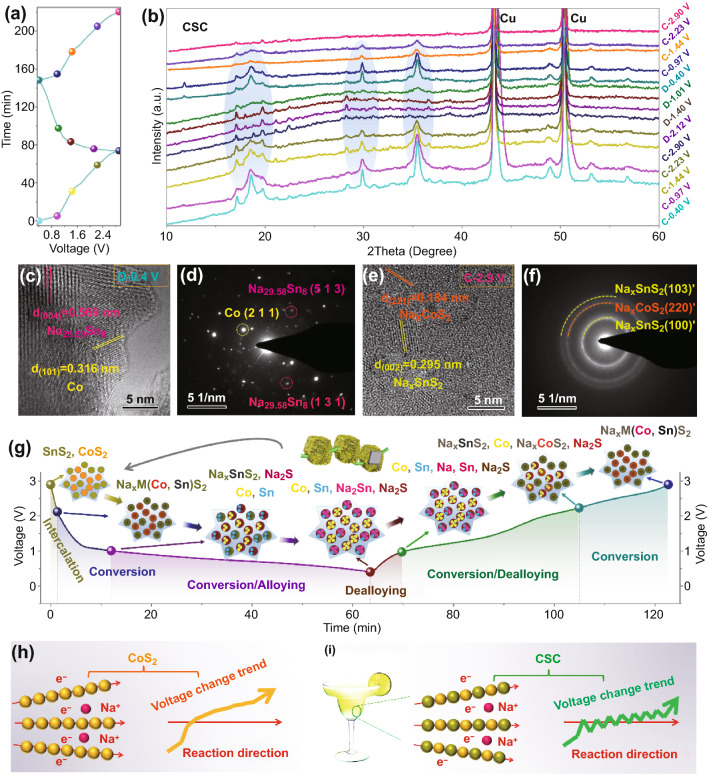
**a** Discharge–charge-time curve and **b** ex situ XRD patterns of CSC anode at different potentials. **c** HR-TEM image and **d** SAED pattern of CSC discharged to 0.4 V. **e** HR-TEM image and **f** SAED pattern of CSC charged to 2.9 V. **g** Schematic illustration of discharge/charge mechanisms of CSC anode. **h** Schematic illustration of reaction route and charge voltage change trend of CoS_2_ anode. **i** Schematic illustration of reaction route and charge voltage change trend of CSC anode, showing cocktail mediation effect among nano-dispersed metal sulfide phases in CSC

To investigate the mechanism that underpins the superior electrochemical behavior of CSC anodes, samples were at various states-of-(dis)charge characterized by ex situ XRD. The copper current collector in a Na//Cu half-cell discharged to 0.4 V shows only the intrinsic diffraction peaks for metallic copper, verifying no evident electrochemical reaction between Na and Cu collector in ether-based electrolyte (Fig. S18). Compared with original samples (CSC, commercial SnS_2_ and CoS_2_), the samples after electrochemical activation exhibit dramatically different XRD patterns, indicating the occurrence of phase transition (Fig. S19). For CoS_2_, the relevant electrochemical reactions are as follows: CoS_2_ + *x*Na^+^  + *x*e^−^ → Na_*x*_CoS_2_, Na_*x*_CoS_2_ + (4 − *x*)Na^+^  + (4 − *x*)e^−^ ↔ 2Na_2_S + Co [60]. For SnS_2_, the corresponding electrochemical reactions are as follows: *x*Na^+^  + SnS_2_ + *x*e^−^ → Na_*x*_SnS_2_, Na_*x*_SnS_2_ + (4-*x*)Na^+^  + (4-*x*)e^−^ ↔ 2Na_2_S + Sn, Sn + yNa^+^  + ye^−^ ↔ Na_y_Sn [40, 61]. Compared with single phases, the CSC anode shows similar featured diffraction peaks to pure SnS_2_, while the peaks from the CoS_2_ diffraction pattern are difficult to discern. This could be associated with differences in crystallinity between products derived from SnS_2_ and CoS_2_. For investigating the mechanism of activated CSC, original CSC anodes were activated for at least 3 cycles to obtain phase-transformed materials. Corresponding to the discharge–charge-time curves in Fig. 4a, the activated CSC anodes at various states-of-charge show repeatable XRD patterns, implying good reversibility during the discharge/charge processes (Fig. 4b). The peak intensity of XRD pattern of anode (such as C-0.97 V, blue) is lower than that of initially charged anode (such as C-0.97 V, pink), which could be associated with the decreased diameter and gradually aggravated amorphization of metal sulfide phases. Similar phenomena have been reported in other metal chalcogenide anodes such as CoSe_2_ and CoS_2_ [13, 60]. At different (dis)charge states, the corresponding XRD of anodes shows different patterns, which should be correlated to the successive formation of different products.

As shown in Fig. 4c, HR-TEM image of CSC discharged to 0.4 V displays interplanar spacings of 0.569 and 0.316 nm, corresponding to lattice plane (0 0 4) of Na_29.58_Sn_8_ and (1 0 1) of Co. Selected area electron diffraction (SAED) patterns reveal the lattice plane (2 1 1) of Co, (5 1 3) and (1 3 1) of Na_29.58_Sn_8_ in the discharged product (Fig. 4d). When charged back to 2.9 V, the crystalline domains in the resulting product are remarkably smaller than those in the discharged state. As shown in Fig. 4e, HR-TEM image of CSC charged to 2.9 V displays interplanar spacings of 0.184 and 0.295 nm, which are assigned to lattice plane of (2 2 1)’ of Na_*x*_CoS_2_ and (0 0 2)’ of Na_*x*_SnS_2_ (with CoS_2_ and SnS_2_ standard phases as reference), respectively. The SAED pattern exhibits typical polycrystalline features, in which lattice plane (2 2 0) of Na_*x*_CoS_2_, (1 0 3) and (1 0 0) of Na_*x*_SnS_2_ can be identified (Fig. 4f). Based on the characterization above, the progress of electrochemical reduction, followed by oxidation, for the CSC electrode is illustrated in Fig. 4g. Typically, SnS_2_ and CoS_2_ phases in CSC experience an initial phase transition to Na^+^-intercalated intermediates (Na_*x*_MS_2_, M = Sn, Co), which act as active materials for subsequent discharge/charge cycles. Based on the analysis above, the exotic property mediation beyond rule-of-mixtures [35, 36] (cocktail mediation effect) among nano-dispersed SnS_2_ and CoS_2_ phases in CSC is schematically illustrated in Fig. 4h–i. Specifically, along the different ionic diffusion directions, the nano-dispersed SnS_2_ and CoS_2_ phases in CSC will alternatively react with Na^+^ carriers, as schematically illustrated in Fig. 4i. The nano-dispersion of SnS_2_ and CoS_2_ phases effectively shortens the ion diffusion path, which can kinetically boost electrochemical processes of both metal sulfide anodes. Due to intrinsic thermodynamics difference, the electrochemical competition is present between SnS_2_ and CoS_2_ phases. Also, it does not exclude one of the two phases could show local kinetic merit owing to the diameter difference between them. Thus, in the CSC anode, the alternative electrochemical reaction processes could coexist between the two phases. It enables complementary charge voltage plateau of different metal sulfide phases, resulting in lowered charge plateau of CSC anode.

### Full-Cell Properties

**Fig. 5:**
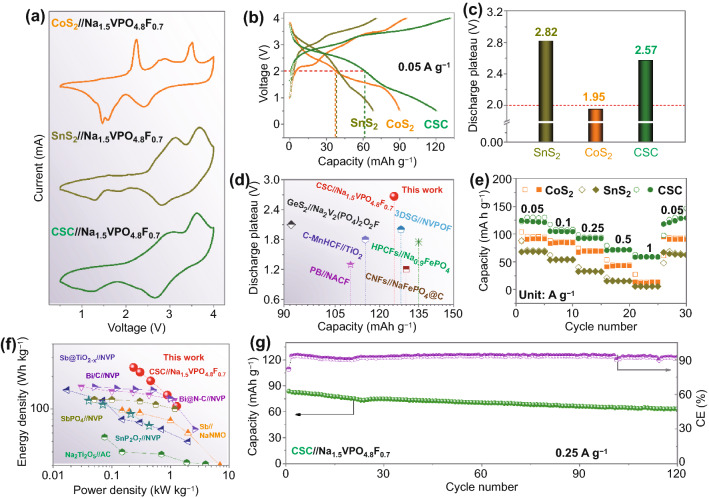
**a** CV curves of CoS_2_//Na_1.5_VPO_4.8_F_0.7_, SnS_2_//Na_1.5_VPO_4.8_F_0.7_, and CSC//Na_1.5_VPO_4.8_F_0.7_ full-cells at 0.5 mV s^−1^. **b** Corresponding discharge/charge curves and **c** discharge plateaus of full-cells at 0.05 A g^−1^. **d** Discharge plateau/capacity comparison of different full-cells. **e** Rate capability of CoS_2_//Na_1.5_VPO_4.8_F_0.7_, SnS_2_//Na_1.5_VPO_4.8_F_0.7_, and CSC//Na_1.5_VPO_4.8_F_0.7_ full-cells. **f** Ragone plots comparison of different full-cells. **g** Long-life cyclability of CSC//Na_1.5_VPO_4.8_F_0.7_ full-cells at 0.25 A g^−1^

To verify the practicability of the CSC anode, a high-voltage cathode material Na_1.5_VPO_4.8_F_0.7_ was employed to assemble CSC//Na_1.5_VPO_4.8_F_0.7_ full-cells. Synthesis of Na_1.5_VPO_4.8_F_0.7_ followed a modified literature method (Supporting Information), and yielded a micro-particle morphology with a well-matched XRD pattern with the standard phase (Fig. S20) [33]. Corresponding to CV curves, Na_1.5_VPO_4.8_F_0.7_ cathode shows *ca.* 3.9 V discharge plateau with low electrochemical polarization, which is suitable for demonstrating the practicability of different anodes (Fig. S21a, b). The Na_1.5_VPO_4.8_F_0.7_ cathode delivers a good rate capability from 0.05 to 0.5 A g^−1^, showing a high reversible capacity of 124.1 mAh g^−1^_electrode_ at 0.05 A/g (Fig. S21c, d). Over 350 cycles at 0.1 A g^−1^, the Na_1.5_VPO_4.8_F_0.7_ cathode shows a capacity of 106.4 mAh g^−1^_electrode_, corresponding to a low capacity decay of 0.02% per cycle (Fig. S22). Figure 5a shows the typical CV curves of CoS_2_//Na_1.5_VPO_4.8_F_0.7_, SnS_2_//Na_1.5_VPO_4.8_F_0.7_, and CSC//Na_1.5_VPO_4.8_F_0.7_ full-cells at 0.5 mV s^−1^. Evidently, the main redox peaks of CoS_2_//Na_1.5_VPO_4.8_F_0.7_ appear at 1.0–2.5 V, implying that its average discharge voltage is in the range. In contrast, the ranges of main redox peaks of SnS_2_//Na_1.5_VPO_4.8_F_0.7_ and CSC//Na_1.5_VPO_4.8_F_0.7_ full-cells are in 2.0–4.0 V, which imply a higher average discharge voltage than that of the former. Figure 5b shows that the discharge capacity available from the CSC//Na_1.5_VPO_4.8_F_0.7_ cell, while the voltage is above 2 V, is *ca.* 61.7 mAh g^−1^_electrode_, which is 1.62 times that of CoS_2_//Na_1.5_VPO_4.8_F_0.7_. As displayed in Fig. 5c, CSC//Na_1.5_VPO_4.8_F_0.7_ full-cells present an average discharge voltage of 2.57 V, which is close to that of SnS_2_//Na_1.5_VPO_4.8_F_0.7_ and *ca.* 0.62 V higher than that with CoS_2_ anode. The CSC anode confers a significantly higher average voltage during discharge of full-cells when compared with CoS_2_ cells. Compared with other full-cells reported previously, CSC//Na_1.5_VPO_4.8_F_0.7_ full-cells also show obvious merits in terms of discharge voltage and capacity (Fig. 5d). Moreover, CSC//Na_1.5_VPO_4.8_F_0.7_ full-cells show a high-rate capability from 0.05 to 1 A g^−1^, delivering a high capacity of 120.3 mAh g^−1^_electrode_ at 0.05 A g^−1^ (Fig. 5e). The corresponding discharge/charge curves are shown in Fig. S23, where the voltage plateaus are well-retained. As exhibited in Fig. 5f, compared with other full-cells with different electrode materials, CSC//Na_1.5_VPO_4.8_F_0.7_ full-cell delivers comparable merits in terms of energy/power density. [62–67] Specifically, ~106.1 Wh kg^−1^_electrode_/1278.3 W kg^−1^_electrode_ are achieved at 1 A g^−1^. When operated over 120 cycles at 0.25 A g^−1^, CSC//Na_1.5_VPO_4.8_F_0.7_ full-cell shows a high capacity of 63.0 mAh g^−1^_electrode_ with a low decay of 0.20% per cycle (Fig. 5g). Such results suggest a good practicability of CSC in full-cells.

## Conclusions

Despite with high-capacity and low-cost merits, the ubiquitous low-rate and high-plateau issues greatly lower the practicability of metal sulfide anodes in full-cells. Herein, enlightened by electrolyte/structure-dependent properties of metal sulfides, CSC anode assembled by nano-dispersed SnS_2_ and CoS_2_ phases is engineered as a case study in ether-based electrolyte, simultaneously realizing high-rate and low-plateau properties. The high nano-dispersity of metal sulfides endows CSC anode with evident cocktail mediation effect similar to high-entropy materials, effectively circumventing intrinsic drawbacks of different metal sulfides. The utilized ether-based electrolyte greatly enhances the reversibility of metal sulfides, sustaining a long-life effectivity of cocktail-like mediation. In half-cells, CSC delivers an ultrahigh-rate capability of 327.6 mAh g^−1^_anode_ at 20 A g^−1^ and remarkably lowered average charge voltage up to *ca.* 0.62 V, far outperforming CoS_2_ phase and SnS_2_/CoS_2_ mixture. The as-assembled CSC//Na_1.5_VPO_4.8_F_0.7_ full-cell shows a good rate capability (0.05–1.0 A g^−1^, 120.3 mAh g^−1^_electrode_ at 0.05 A g^−1^) and a high average discharge voltage up to 2.57 V, comparable to full-cells with alloy-type anodes. Kinetics and mechanism studies further verify that the cocktail-like mediation effect largely boosts charge transfer and ionic diffusion in CSC, while alternative and complementary electrochemical processes between different nano-dispersed metal sulfides (SnS_2_ and CoS_2_) and Na^+^ carriers account for the lowered charge plateau of CSC. This work shows a unique electrolyte/structure-dependent cocktail-like mediation effect of metal sulfide anodes, which will boost the development of high-rate/-voltage sodium-ion full batteries.

## Supplementary Information

Below is the link to the electronic supplementary material.

Supplementary file1 (PDF 3447 kb)
