# Supplementary material for: Electrolyte/Structure-Dependent Cocktail Mediation Enabling High-Rate/Low-Plateau Metal Sulfide Anodes for Sodium Storage
Source: Nanomicro Lett. 2021 Aug 17;13:178. doi: 10.1007/s40820-021-00686-4 (PMC8371071; doi:10.1007/s40820-021-00686-4)
Supplement: Supplementary file 1 — Supplementary file1 (PDF 3447 kb) [file 40820_2021_686_MOESM1_ESM.pdf]

Supporting Information for

Electrolyte/Structure-Dependent Cocktail Mediation Enabling High-Rate/Low-Plateau Metal Sulfide Anodes for Sodium Storage

Yongchao Tang[1, 2, #], Yue Wei[3, #], Anthony F. Hollenkamp[2, *], Mustafa Musameh[2], Aaron Seeber[2], Tao Jin[2, 4], Xin Pan[1], Han Zhang[1], Yanan Hou[1], Zongbin Zhao[1], Xiaojuan Hao[2, *], Jieshan Qiu[1, *], Chunyi Zhi[5]

[1]State Key Lab of Fine Chemicals, Liaoning Key Lab for Energy Materials and Chemical Engineering, School of Chemical Engineering, Dalian University of Technology, Dalian 116024, P. R. China

[2]Manufacturing, Commonwealth Scientific and Industrial Research Organization (CSIRO), Clayton, VIC 3168, Australia

[3]State Key Laboratory of Fine Chemicals, School of Chemical Engineering, Dalian University of Technology, Dalian 116024, P. R. China

[4]School of Resources and Environment Engineering, Shandong agriculture and engineering university, Jinan, 250100, P. R. China

[5]Department of Materials Science and Engineering, City University of Hong Kong, 83 Tat Chee Avenue, Hong Kong, P. R. China

[#]Yongchao Tang and Yue Wei contributed equally to this work.

*Corresponding authors. E-mail: Tony.Hollenkamp@csiro.au (Anthony F. Hollenkamp); Xiaojuan.Hao@csiro.au (Xiaojuan Hao); jqiu@dlut.edu.cn (Jieshan Qiu)

S1 Experiment Section

S1.1 Chemicals

All chemicals were used as received without further purification except acidized carbon nanotubes. Cobalt nitrate hexahydrate ($Co(NO_3)_2{\cdot}6H_2O$ > 98%), 2-methylimidazole ($C_4H_6N_2$ > 99%), polyvinylpyrrolidone (PVP, MW 40 000), and tin dioxides (SnO_2, 99.9%) were purchased from Sigma Aldrich; Methanol (99.8%) was obtained from Alfa Aesar. Vanadium pentoxide (V_2O_5, 99%), ammonium dihydrogen phosphate ($NH_4H_2PO_4$, 99%), sodium fluoride (NaF, 99%), and sodium carbonate (Na_2CO_3, 99%) were purchased from Aladdin. Acidized carbon nanotubes were prepared following the previous method [S1].

S1.2 Synthesis of Ammonium Thiostannate Solution ($(NH_4)_4Sn_2S_6$)

SnS_2 was freshly synthesized by sulfurizing 0.753 g SnO_2 with 1.502 g thioacetamide at 180 °C for 12 h. In a typical run for preparing $(NH_4)_4Sn_2S_6$ solution, 0.319 g SnS_2

was dispersed in 30 mL ammonium sulfide (3 mL, 40~48 wt% in deionized water (27 mL)) solution under shaking, and then hydrothermally treated at 180 °C for 12 h.

S1.3 Synthesis of Acidized Carbon Nanotubes-stringed ZIF-67 (C-ZIF-67)

Typically, 25 mg acidized CNTs, 0.305 g PVP (MW 40 000), and 0.873 g $Co(NO_3).6H_2O$ was first dispersed into 30 mL methanol by 30 min ultra-sonication, then kept stirring for 30 min to form solution A; subsequently, 5 mL 2-methylimidazole/methanol solution (containing 0.984 g 2-methylimidazole) was slowly added into solution A, after stirring for another 30 min, kept incubating for 24 h at 25 °C. For comparison, ZIF-67 particles were synthesized under similar conditions without addition of acidized CNTs.

S1.4 Synthesis of Carbon Nanotubes-stringed Metal Sulfides Ultrastructure (CSC)

For obtaining CSC, 5 mL $(NH_4)_4Sn_2S_6$ solution was rapidly added to 30 mL ethanol suspension containing 30 mg C-ZIF-67, after rigorously shaking around 30 seconds, the resulting mixture was cooled in an ethanol-bath to different temperatures (0, -5, and -10 °C) for 1 h. To probe the formation of CoS_2-SnS_2 heteroassemblies, different dosages (3, 4, 5, 7 mL) of $(NH_4)_4Sn_2S_6$ solution were added for the reaction. Subsequently, such mixtures were centrifuged/washed with ethanol. As-obtained samples were vacuum-dried and annealed at 450 °C for 3 h under a constant flow of N_2. For comparison, CoS_2@SnS_2 was similarly synthesized by using 30 mg ZIF-67 instead of C-ZIF-67.

S1.5 Synthesis of $Na_{1.5}VPO_{4.8}F_{0.7}$ Cathode

Typically, 1.819 g V_2O_5 and 2.30 g $NH_4H_2PO_4$ were blended by ball milling for 24 h, and the resulting mixture was annealed at 750 °C for 4 h in air to obtain $VOPO_4$ powder. To synthesize VPO_4 powder was synthesized by annealing stoichiometric amount of V_2O_5 and $NH_4H_2PO_4$, and 20 mol% super P mixture (ball-milled for 24 h) at 850 °C for 2 h in Ar flow. Subsequently, $VOPO_4$, VPO_4, NaF, and Na_2CO_3 precursors were ball-milled for 24 h with a molar ratio of 8:2:7:4. The resulting mixture was annealed at 750 °C for 1.5 h in Ar flow, forming gray $Na_{1.5}VPO_{4.8}F_{0.7}$ powder.

S1.6 Characterization of the Materials

The crystal structure of samples was characterized by X-ray diffraction using either a Bruker D8 Advance Series 1 or a Rigaku D/Max 2400 diffractometer, both operating under Cu Kα radiation ($\lambda \approx 1.5406$ Å). The morphology was analyzed by field emission scanning electron microscopy (FE-SEM). Structural details were further characterized by transmission electron microscopy (TEM, FEI Tecnai G2 Spirit, operated at 120 kV) and high-resolution transmission electron microscopy (HR-TEM, FEI Tecnai G2 F30S-Twin, operated at 300 kV). Selected area electron diffraction (SAED) patterns were collected with a Gatan charge-coupled device (CCD) camera in a digital format. Elemental content was tested by inductively coupled plasma-mass spectrometry (ICP-MS, Agilent 7500cx). The N_2 adsorption/desorption test of materials was conducted at

77 K with Micromeritics ASAP 2020 apparatus. The surficial properties of samples were collected by X-ray photoelectron spectroscopy (XPS, Thermo Scientific K-Alpha).

S1.7 Electrochemical Performance Test

The electrodes were fabricated by mixing different samples, acetylene carbon black, and binder (poly (vinylidene difluoride), PVDF) with the weight ratio of 7 : 2 : 1 in sufficient N-methyl-2-pyrrolidone (NMP) solvent to form a slurry suitable for coating with a doctor blade on to sheets of copper foil. After vacuum-drying at 100 °C overnight, disk electrodes were punched out and weighted. The mass loadings of anode and cathode was controlled to 0.6~0.9 and 3.6~5.4 mg cm^{-2}, respectively. CR2016 coin cells were assembled in an argon-filled glove box (UniLab, Mbraun, Germany). In a typical half-cell assembly, Na foil (Sigma-Aldrich, 99%) and glass microfiber (Whatman) were used as the counter electrode and separator, respectively. The used ether-based electrolyte was 1 M $NaPF_6$ in 1/1 (volumetric ratio) of dimethoxyethane (DME) and diethylene glycol dimethyl ether (Diglyme). The used ester-based electrolyte was 1 M $NaPF_6$ in 1/1 (volumetric ratio) of ethylene carbonate (EC) and propene carbonate (PC) with 5% fluoroethylene carbonate (FEC). Cyclic voltammetry (CV) and discharge/charge tests were carried out on a BioLogic electrochemical workstation (VMP 3B-5) and Land battery tester, respectively. GITT tests were conducted at a pulsed current density of 0.05 A g^{-1} for 30 min, followed by 2-h rest. The full cell was assembled with peactivated anodes and $Na_{1.5}VPO_{4.8}F_{0.7}$ cathode, in which mass loading ratio of anode to cathode was 1/6. The capacity of full cell was calculated based on total electrode.

S2 Supplementary Tables and Figures

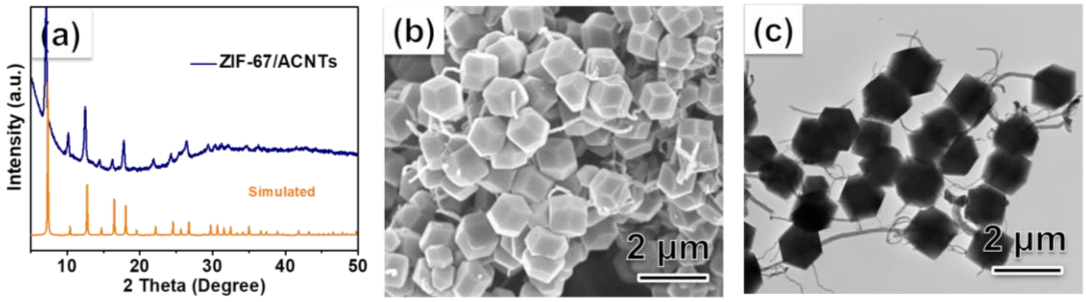

Fig. S1 Morphology and composition characterization: **a** XRD pattern of ZIF-67/CNTs. **b-c** SEM and TEM images of ZIF-67/CNTs showing the ZIF-67 particles well-stringed by CNTs

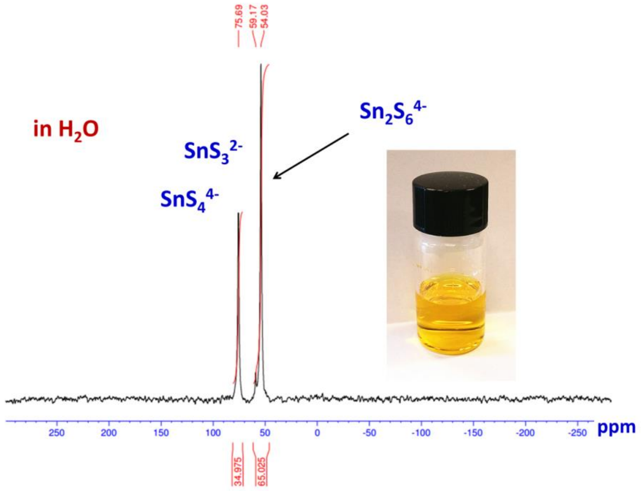

Fig. S2 Optical image (inset) and Sn^{119} NMR spectrum of ammonium thiostannate solution

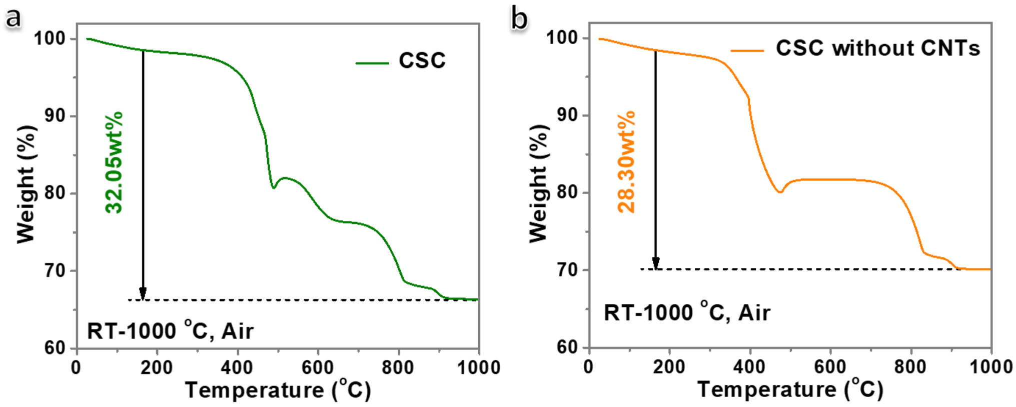

Fig. S3 TGA profiles of samples (**a** CSC, **b** CSC without CNTs) conducted from room temperature to 1000 °C in air, showing a CNTs content of 3.75wt%

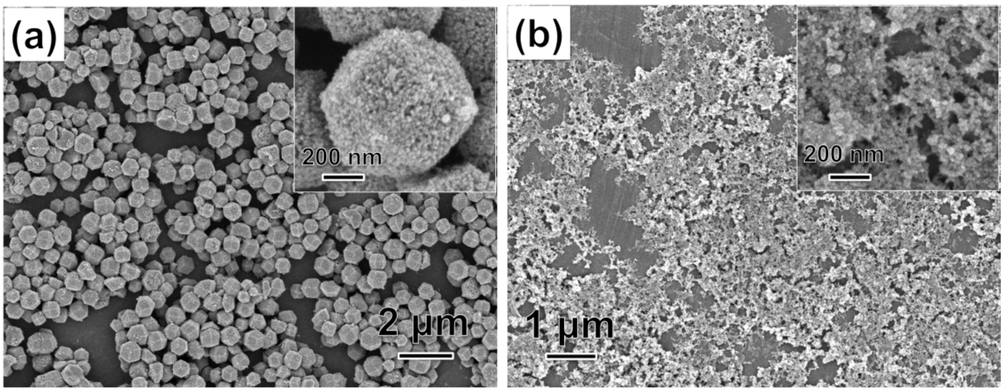

Fig. S4 The effect of precursors in ethanol solvent on material structure: **a** FE-SEM images of material obtained using ZIF-67 to react with $(NH_4)_2S$ solution. **b** FE-SEM images of material obtained using $Co(NO_3)_2$ to react with ammonium thiostannate solution

As shown in **Fig. S4a**, the resulting materials inherit the polyhedral structure from ZIF-67 but without outer sheets, indicating that the formation of outer sheets is triggered by thiostannate species rather than S^{2-}. Alternatively, when employing $Co(NO_3)_2$ to react with thiostannate species, only heteromorphic nanoparticles were found instead of radiation-like heteroassemblies (**Fig. S4b**). Thus ZIF-67 actually functions as a conformal substrate to control the release of Co^{2+} from ligand-bound sites, affording the formation of the outer sheets.

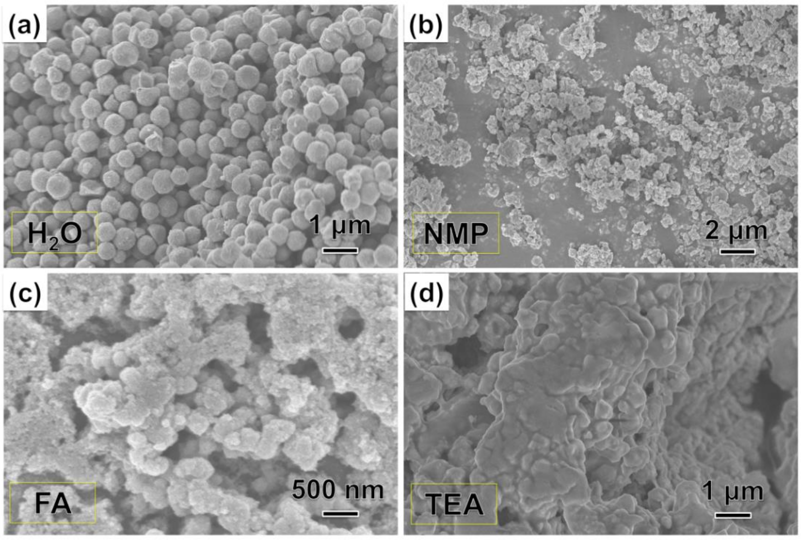

Fig. S5 SEM images of materials prepared in different solvents: **a** Deionized water. **b** 1-methyl-2-pyrrolidinone (NMP). **c** Formamide (FA). **d** Triethanolamine (TEA)

The FE-SEM images in **Fig. S5** exhibit the striking impact of different solvents including deionized water, NMP, formamide, and triethanolamine on material structures. Except for in deionized water, the polyhedral structure from ZIF-67 is hardly sustained in other three solvents. This effect is most likely due to stability differences of ion-exchanged intermediates in the respective solvents.

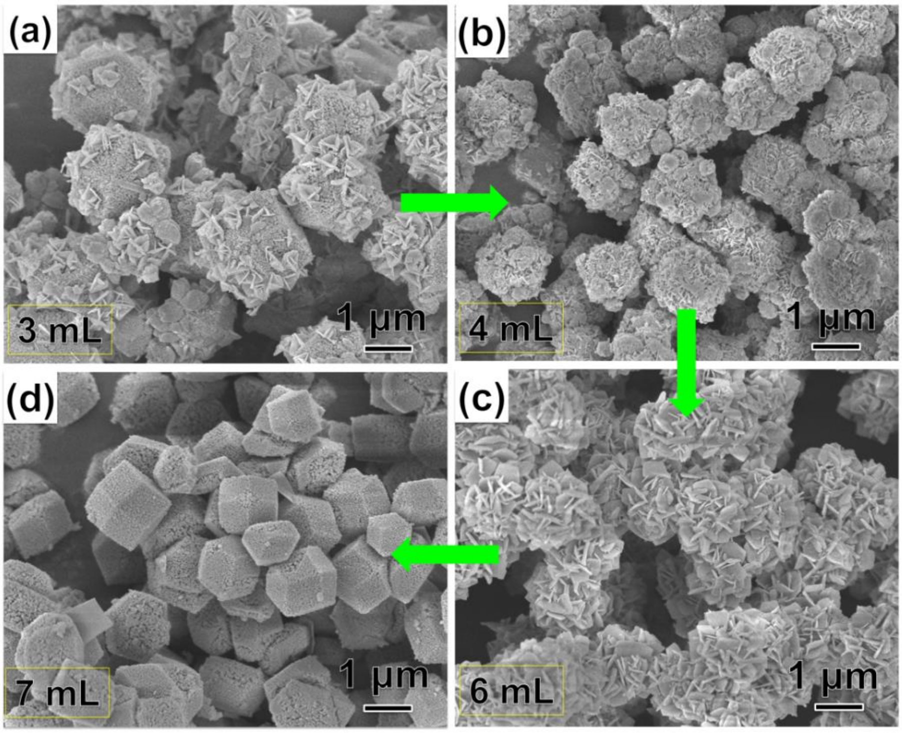

Fig. S6 Effect of thiostannate species dosages: **a** 3 mL, **b** 4 mL, **c** 6 mL, and **d** 7 mL on material structures

Another factor that became apparent during the development of the synthesis was the vital role played by the dosage of thiostannate in building the unique heteroassemblies. As shown in the FE-SEM images of **Fig. S6a-b**, the outer nanosheets exhibit self-assembly behavior on the polyhedral surface, and the number of nanosheets increases with the increase in dosage from 3 to 4 mL; when the dosage increases to 5 mL, well-defined CSC was obtained (**Fig. 1e**). With further increase in dosage to 6 mL, an ordered arrangement of outer nanosheets is no longer possible, due to the limited core surface (**Fig. S6c**). When 7 mL thiostannate solution is added, the outer nanosheets cannot form (**Fig. S6d**). Evidently, the concentration of thiostannate ($C_{Sn_xS_y^{n-}}$) is crucial for the formation of outer nanosheets. When Co^{2+} concentration ($C_{Co^{2+}}$) is $\geq C_{Sn_xS_y^{n-}}$, the outer nanosheets form; if $C_{Co^{2+}}$ is $< C_{Sn_xS_y^{n-}}$, the outer nanosheets fall off due to the fast erosion of core surface preventing the nucleation of outer nanosheets. Thus, by mediating the temperature and concentration, CSC ultrastructure with vertical and small outer nanosheets can be readily obtained for short ion diffusion.

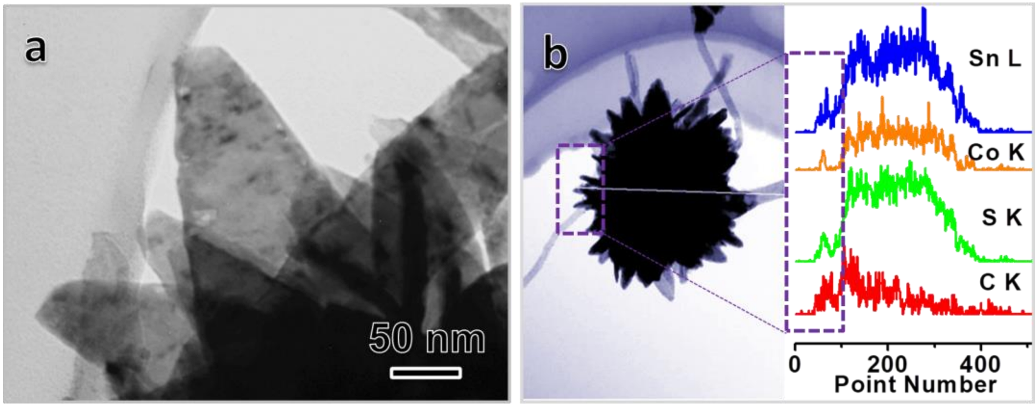

Fig. S7 a TEM image of CSC showing the morphology of shell composed of sheets. **b** TEM-line scan profiles of the shell of CSC

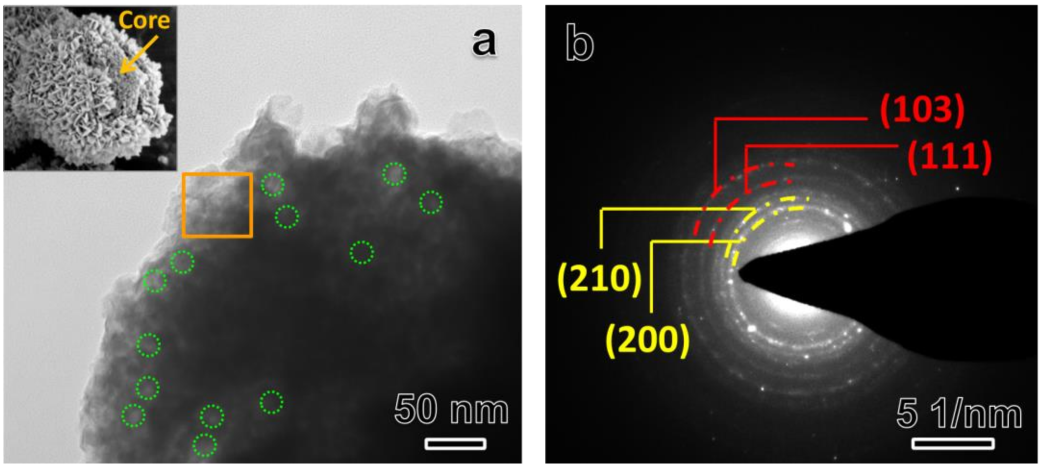

Fig. S8 a TEM image of CSC showing the morphology of core corresponding to the SEM image (inset). **b** SAED pattern corresponding to the boxed part in **a** showing the presence of CoS_2 (yellow), and SnS_2 (red)

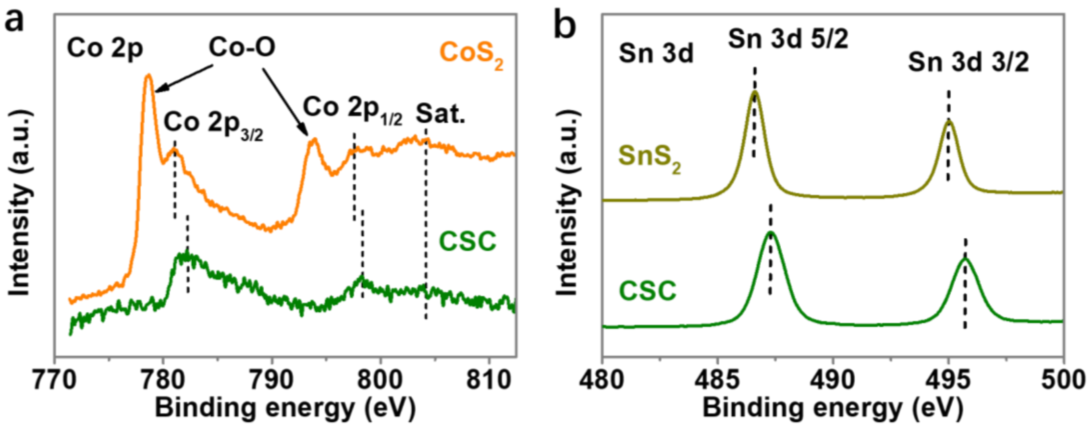

Fig. S9 a High-resolution XPS of Co 2p of CSC and commercial CoS_2 samples.

b High-resolution XPS of Sn 3d of CSC and commercial SnS_2 samples

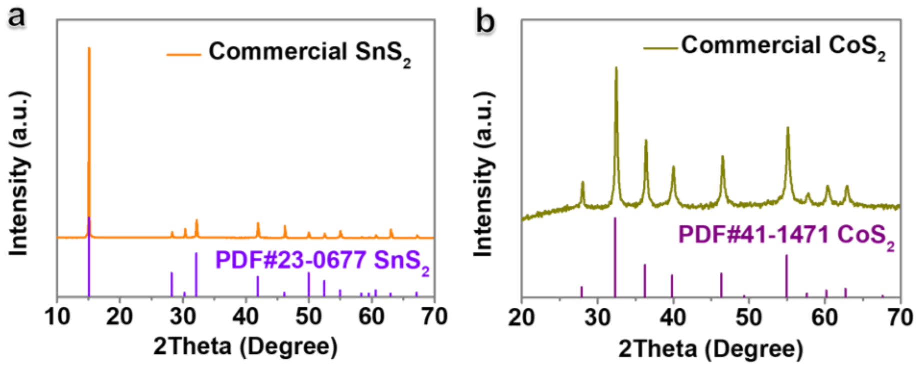

Fig. S10 XRD patterns of **a** commercial SnS_2 and **b** commercial CoS_2 samples

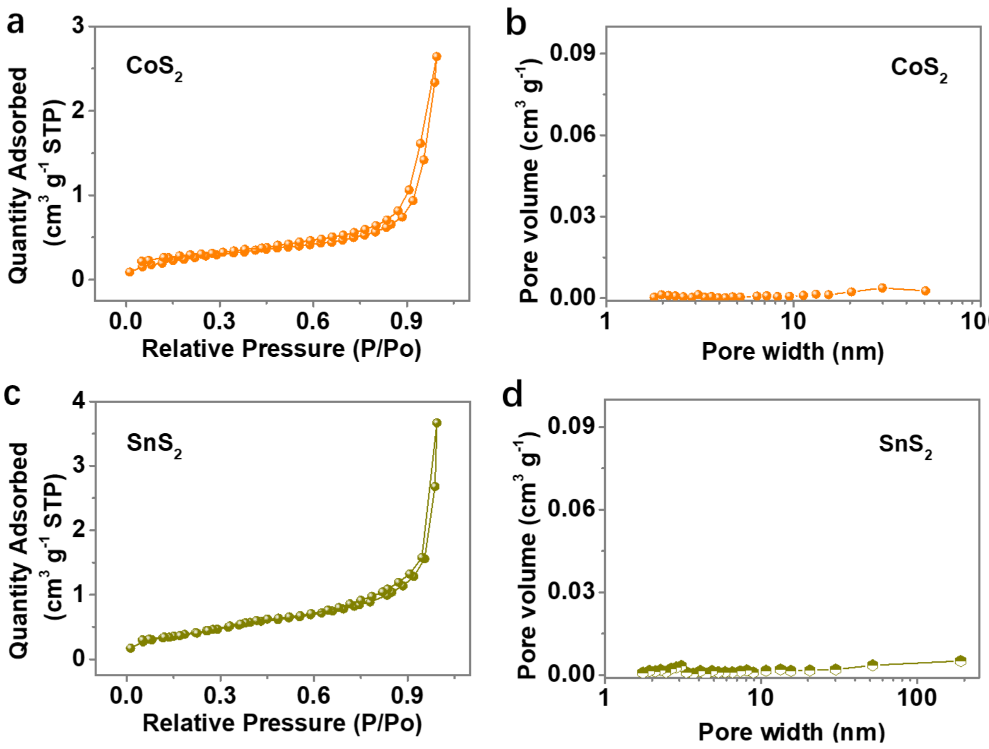

Fig. S11 a N_2 isotherms and **b** corresponding pore width distribution of commercial CoS_2 powders. **c** N_2 isotherms and **d** corresponding pore width distribution of commercial SnS_2 powders.{Wang, 2018 #2086}

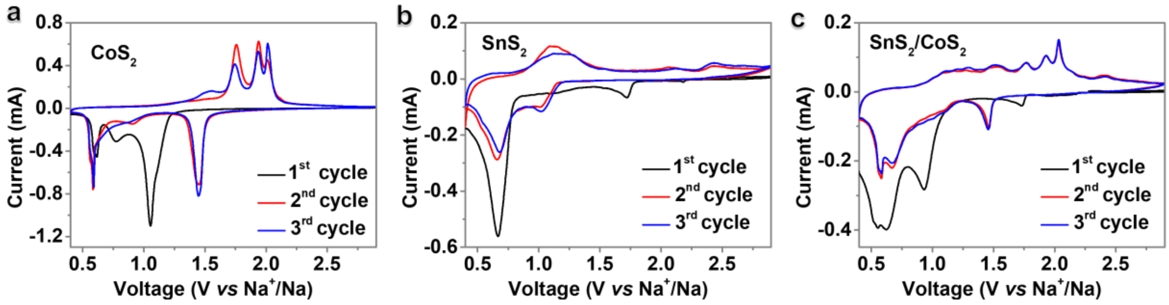

Fig. S12 CV curves of **a** Na//CoS_2, **b** Na//SnS_2, and **c** Na//SnS_2/CoS_2 half cells

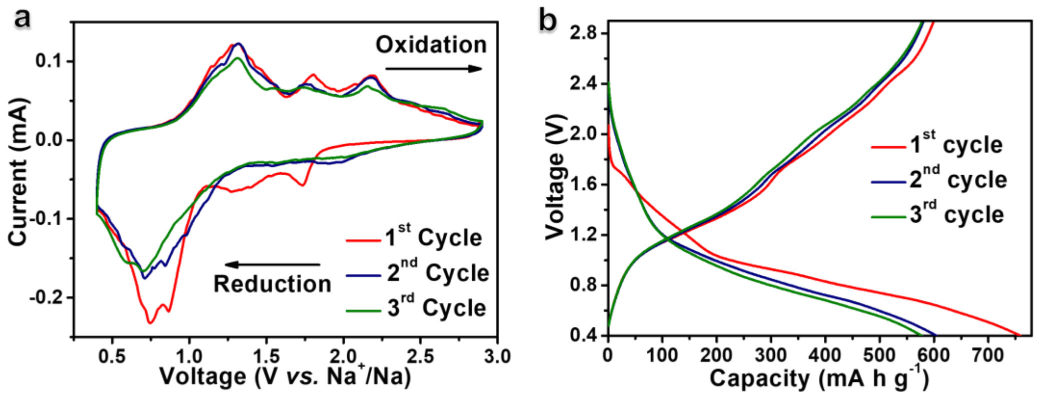

Fig. S13 a CV curves at 0.1 mV s^{-1} and **b** discharge/charge curves (at 0.5 A g^{-1}) of Na//CSC half cell with ether-based electrolyte

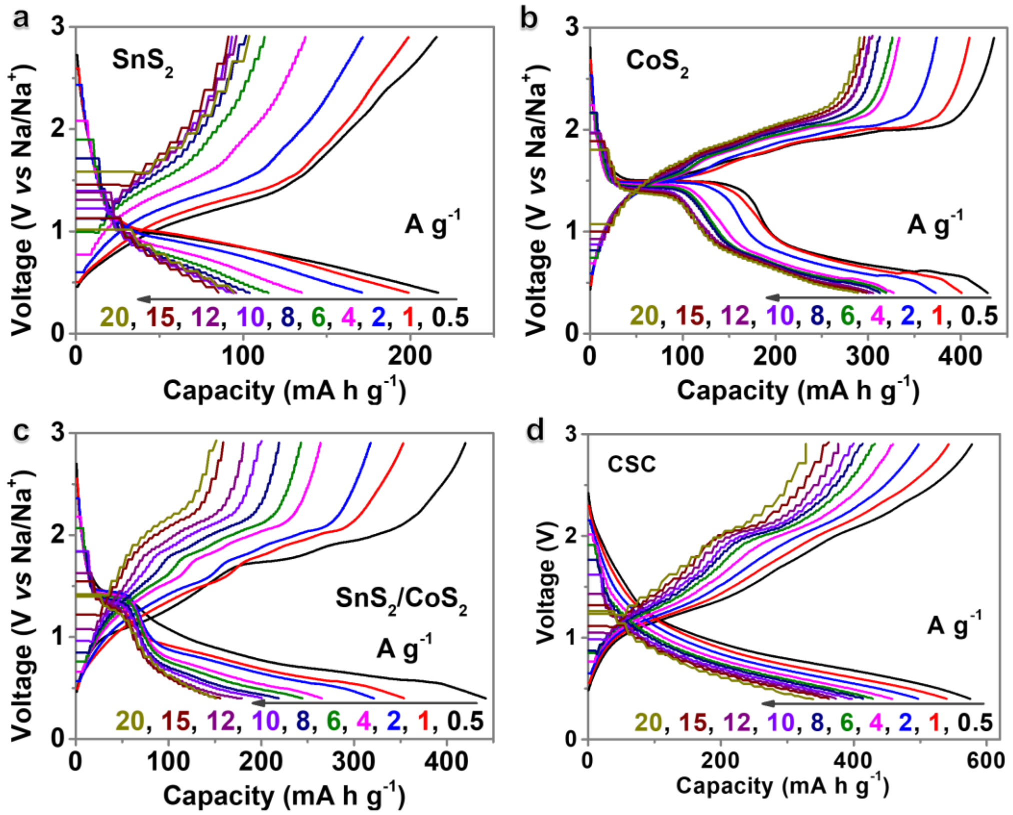

Fig. S14 Charge-discharge profiles of **a** SnS_2, **b** CoS_2, **c** SnS_2/CoS_2 mixture, and **d** CSC anodes at different current densities

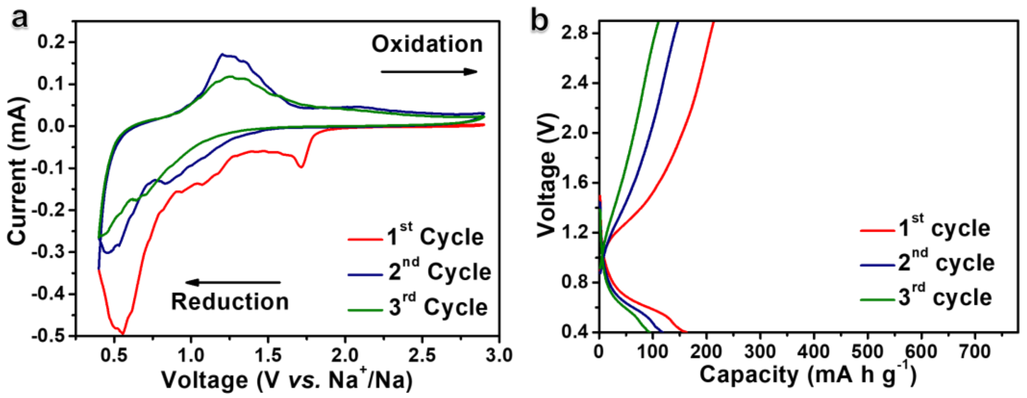

Fig. S15 a CV curves at 0.1 mV s^{-1} and **b** discharge/charge curves (at 0.5 A g^{-1}) of Na//CSC half cells in ester-based electrolytes

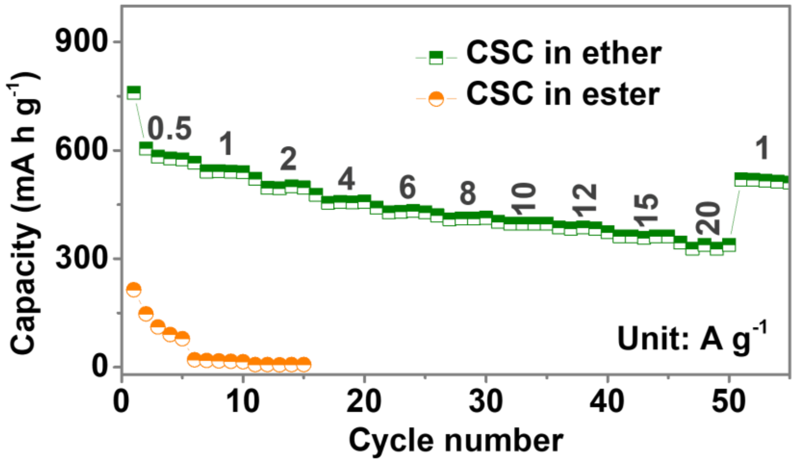

Fig. S16 Rate capability comparison of Na//CSC half cell in ether- and ester-based electrolytes

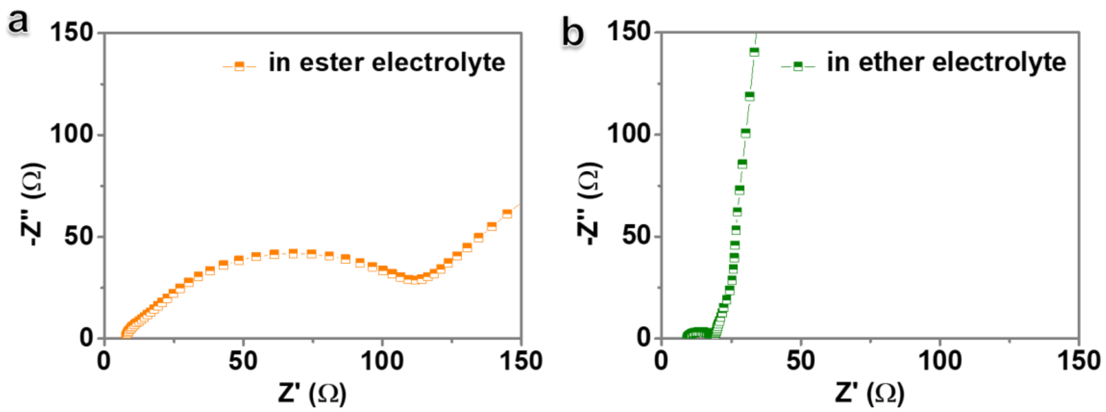

Fig. S17 Nyquist curves of Na//CSC half cells in **a** ester-based and **b** ether-based electrolytes

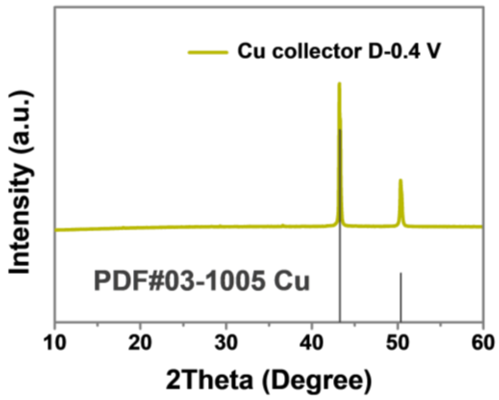

Fig. S18 XRD patterns of Cu foil (collector) as anode (control sample) discharged to 0.4 V, showing no obvious electrochemical reaction between Cu and Na metal

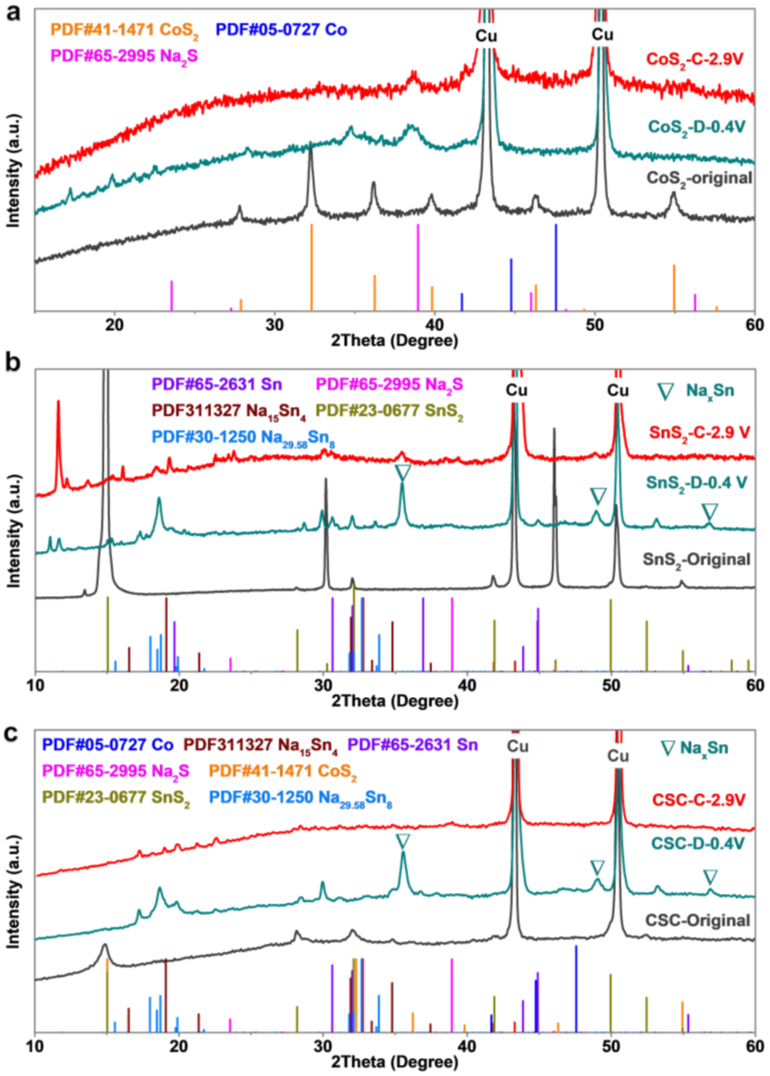

Fig. S19 XRD patterns of anodes at different discharge/charge states: **a** Original CoS_2, CoS_2 discharged to 0.4 V and charged to 2.9 V. **b** Original SnS_2, SnS_2 discharged to 0.4 V and charged to 2.9 V. **c** Original CSC, CSC discharged to 0.4 V and charged to 2.9 V

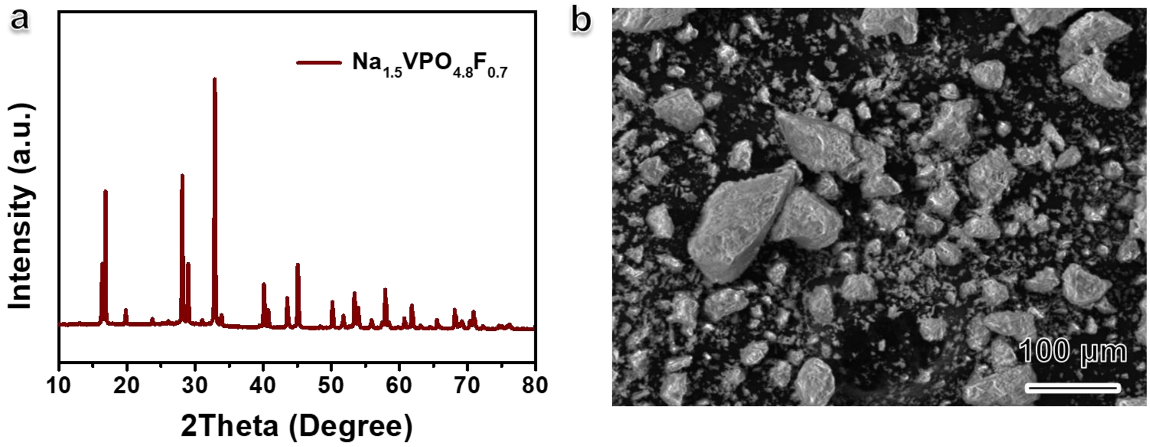

Fig. S20 a XRD pattern and **b** SEM image of the synthesized $Na_{1.5}VPO_{4.8}F_{0.7}$ phase

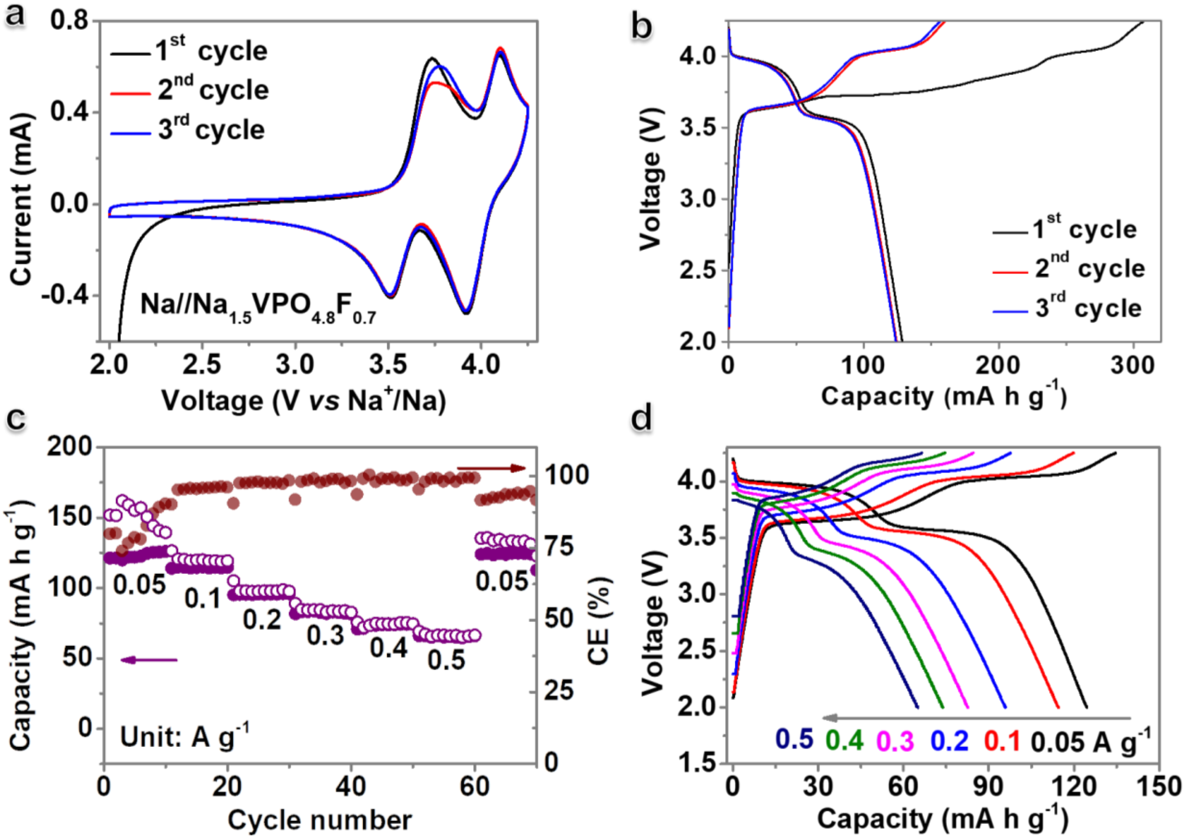

Fig. S21 a Initial CV curves at 0.1 mV s^{-1} and **b** corresponding discharge/charge curves of Na//$Na_{1.5}VPO_{4.8}F_{0.7}$ half cell. **c** Rate capability and **d** corresponding discharge/charge curves of Na//$Na_{1.5}VPO_{4.8}F_{0.7}$ half cell

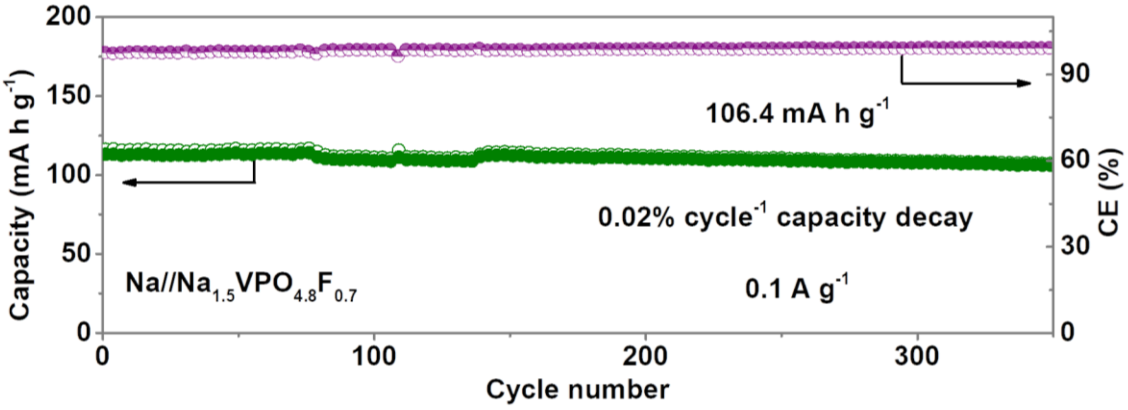

Fig. S22 Long-life cyclability of Na//$Na_{1.5}VPO_{4.8}F_{0.7}$ half cell at 0.1 A g^{-1}

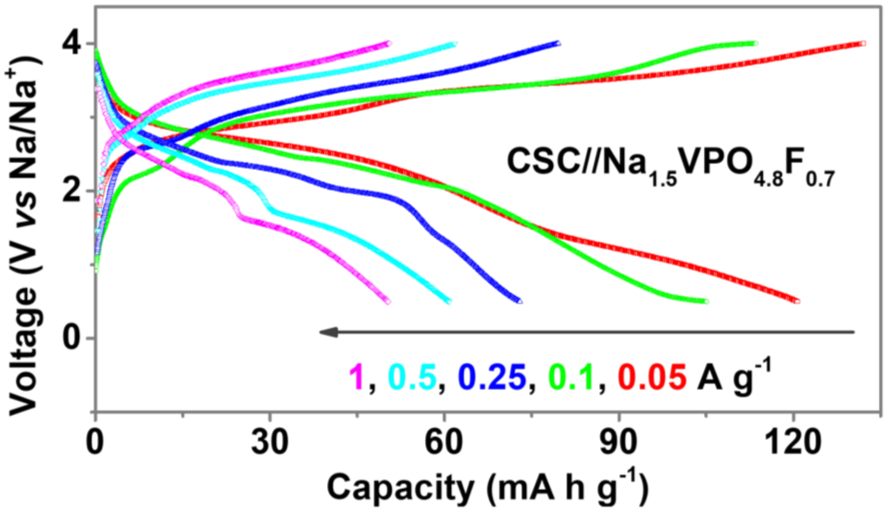

Fig. S23 Discharge/charge curves of CSC//$Na_{1.5}VPO_{4.8}F_{0.7}$ full cell at different current densities

Table S1 Inductively coupled plasma-mass spectrometry (ICP-MS) analysis on the mass content of SnS_2 and CoS_2 in the CSC sample

Sample	Mass (g)	Vo (mL)	Elements	Content (mg kg^{-1})	Atomic ratio (*vs* Co)
CSC	0.0399	25	Co	93971.8	1
	0.0399	25	S	278328	1.73
	0.0399	25	Sn	327774	5.46

Table S2 Rate capability comparison of different metal chalcogenide-based anodes [S2-S9]

Current density	Our work	VS_4	$NiCo_2S_4$	Co_3S_4@1TMoS_2/NC-L	Cu_2MoS_4	CoZn-Se	CoSe@CSs	CoZn–Se_2@NC	Co_9S_8/ZnS
	Specific capacity (mAh g^{-1})								
0.05				619	259.5				
0.1		608			250	425	900	510	525
0.2		552	560.5	557	240	402	711	470	475
0.5	576.4	471		517	230	375	644	403	430
0.8								380	
1	540.4	422	505.8	480	220	352	584	372	405
1.4								340	
1.8								304	
2	498.9	340		438	195	330	535	292	365
2.5									
4	461		448.1			305			320
5		238		338	135		390		
6	434.9		416.2			282			295
7.5									
8	414					270	286		250
10	400.3	173				263			240
12	382.9								
15	362.1								
20	327.6	123							

Table S3 Ragone plots comparing the energy density and power density of different full cells [S10-S15]

**This work**		Sb//NaNMO		Bi@N-C//NVP		$Sb@TiO_{2-x}$//NVP		Bi/C//NVP		$Na_2Ti_2O_5$//AC		SnP_2O_7//NVP		$SbPO_4$//NVP	
P	E	P	E	P	E	P	E	P	E	P	E	P	E	P	E
0.2345	242.7	7	30	0.03	160	0.017	150	0.05	160	0.075	55	0.04	120	0.05	123
0.3029	219	2	60	0.1	146	0.07	120	0.12	155	0.15	40	0.07	110	0.1	122

0.4582	181.9	1	78	0.23	143	0.15	80	0.25	152	0.7	38	0.21	90	0.3	118
0.8815	134.9	0.3	92	0.43	135	0.3	75	0.5	150	1.9	32	0.43	70	0.56	110
1.2783	106.1	0.2	95	0.95	125	0.7	65	1	125	4	31			1.19	100
		0.15	97	1.15	120	2	50	2.6	65						

P = Power density (kW kg^{-1}), E = Energy density (Wh kg^{-1}).

Supplementary References

[S1] Y. Liu, T.C. Hughes, B.W. Muir, L.J. Waddington, T.R. Gengenbach et al., Water-dispersible magnetic carbon nanotubes as T_2-weighted MRI contrast agents. Biomaterials **35**(1), 378-386 (2014). https://doi.org/10.1016/j.biomaterials.2013.09.079

[S2] S. Wang, F. Gong, S. Yang, J. Liao, M. Wu et al., Graphene oxide-template controlled cuboid-shaped high-capacity VS_4 nanoparticles as anode for sodium-ion batteries. Adv. Funct. Mater. **28**(34), 1801806 (2018). https://doi.org/10.1002/adfm.201801806

[S3] S. Li, P. Ge, F. Jiang, H. Shuai, W. Xu et al., The advance of nickel-cobalt-sulfide as ultra-fast/high sodium storage materials: The influences of morphology structure, phase evolution and interface property. Energy Storage Mater. **16**, 267-280 (2019). https://doi.org/10.1016/j.ensm.2018.06.006

[S4] Y. Li, R. Zhang, W. Zhou, X. Wu, H. Zhang et al., Hierarchical MoS_2 hollow architectures with abundant Mo vacancies for efficient sodium storage. ACS Nano **13**(5), 5533-5540 (2019). https://doi.org/10.1021/acsnano.9b00383

[S5] J. Chen, L. Mohrhusen, G. Ali, S. Li, K.Y. Chung et al., Electrochemical mechanism investigation of Cu_2MoS_4 hollow nanospheres for fast and stable sodium ion storage. Adv. Funct. Mater. **29**(7), 1807753 (2019). https://doi.org/10.1002/adfm.201807753

[S6] G. Fang, Q. Wang, J. Zhou, Y. Lei, Z. Chen et al., Metal organic framework-templated synthesis of bimetallic selenides with rich phase boundaries for sodium-ion storage and oxygen evolution reaction. ACS Nano **13**(5), 5635-5645 (2019). https://doi.org/10.1021/acsnano.9b00816

[S7] Z. Ali, T. Tang, X. Huang, Y. Wang, M. Asif et al., Cobalt selenide decorated carbon spheres for excellent cycling performance of sodium ion batteries. Energy Storage Mater. **13**, 19-28 (2018). https://doi.org/10.1016/j.ensm.2017.12.014

[S8] Y. Jiang, G. Zou, H. Hou, J. Li, C. Liu et al., Composition engineering boosts

voltage windows for advanced sodium-ion batteries. ACS Nano **13**(9), 10787-10797 (2019). https://doi.org/10.1021/acsnano.9b05614

[S9] G. Fang, Z. Wu, J. Zhou, C. Zhu, X. Cao et al., Observation of pseudocapacitive effect and fast ion diffusion in bimetallic sulfides as an advanced sodium-ion battery anode. Adv. Energy Mater. **8**(19), 1703155 (2018). https://doi.org/10.1002/aenm.201703155

[S10] L. Liang, Y. Xu, C. Wang, L. Wen, Y. Fang et al., Large-scale highly ordered Sb nanorod array anodes with high capacity and rate capability for sodium-ion batteries. Energy Environ. Sci. **8**(10), 2954-2962 (2015). https://doi.org/10.1039/C5EE00878F

[S11] P. Xue, N. Wang, Z. Fang, Z. Lu, X. Xu et al., Rayleigh-instability-induced bismuth nanorod@nitrogen-doped carbon nanotubes as a long cycling and high rate anode for sodium-ion batteries. Nano Lett. **19**(3), 1998-2004 (2019). https://doi.org/10.1021/acs.nanolett.8b05189

[S12] N. Wang, Z. Bai, Y. Qian, J. Yang. Double-walled Sb@TiO_{2-x} nanotubes as a superior high-rate and ultralong-lifespan anode material for Na-ion and Li-ion batteries. Adv. Mater. **28**(21), 4126-4133 (2016). https://doi.org/10.1002/adma.201505918

[S13] J. Pan, S. Chen, Q. Fu, Y. Sun, Y. Zhang et al., Layered-structure $SbPO_4$/reduced graphene oxide: An advanced anode material for sodium ion batteries. ACS Nano **12**(12), 12869-12878 (2018). https://doi.org/10.1021/acsnano.8b08065

[S14] Q. Gui, D. Ba, Z. Zhao, Y. Mao, W. Zhu et al., Synergistic coupling of ether electrolyte and 3D electrode enables titanates with extraordinary coulombic efficiency and rate performance for sodium-ion capacitors. Small Methods **3**(2), 1800371 (2019). https://doi.org/10.1002/smtd.201800371

[S15] H. Yuan, F. Ma, X. Wei, J.-L. Lan, Y. Liu et al., Ionic-conducting and robust multilayered solid electrolyte interphases for greatly improved rate and cycling capabilities of sodium ion full cells. Adv. Energy Mater. **10**(37), 2001418 (2020). https://doi.org/10.1002/aenm.202001418
